# Supplementary material for: A fitness distribution law for amino-acid replacements
Source: bioRxiv. 2024 Oct 15:2024.10.11.617952. Preprint. [Version 1] doi: 10.1101/2024.10.11.617952 (PMC11507765; doi:10.1101/2024.10.11.617952)
Supplement: 1 [file NIHPP2024.10.11.617952V1-supplement-1.pdf]

bioRxiv preprint doi: https://doi.org/10.1101/2024.10.11.617952; this version posted October 15, 2024. The copyright holder for this preprint (which was not certified by peer review) is the author/funder, who has granted bioRxiv a license to display the preprint in perpetuity. It is made available under aCC-BY 4.0 International license.

Supporting information

bioRxiv preprint doi: https://doi.org/10.1101/2024.10.11.617952; this version posted October 15, 2024. The copyright holder for this preprint (which was not certified by peer review) is the author/funder, who has granted bioRxiv a license to display the preprint in perpetuity. It is made available under aCC-BY 4.0 International license.

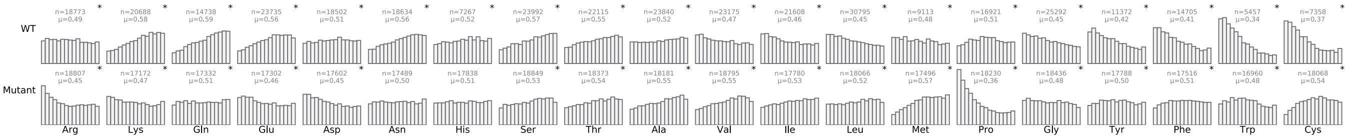

Fig. S1. Quantile distributions of fitness effects from ProteinGym, categorized by wild-type (“from”) and mutant (“to”) amino acids. The distributions in the top row aggregate all 358,080 mutational effects by the wild-tpe amino acid, and those in the bottom row aggregate the same mutational effects by the mutant amino acid. Distributions that are significantly different from a uniform distribution are marked with a star in the upper-right corner. The sample size for each distribution is indicated by n, and the mean of each distribution is indicated by μ.

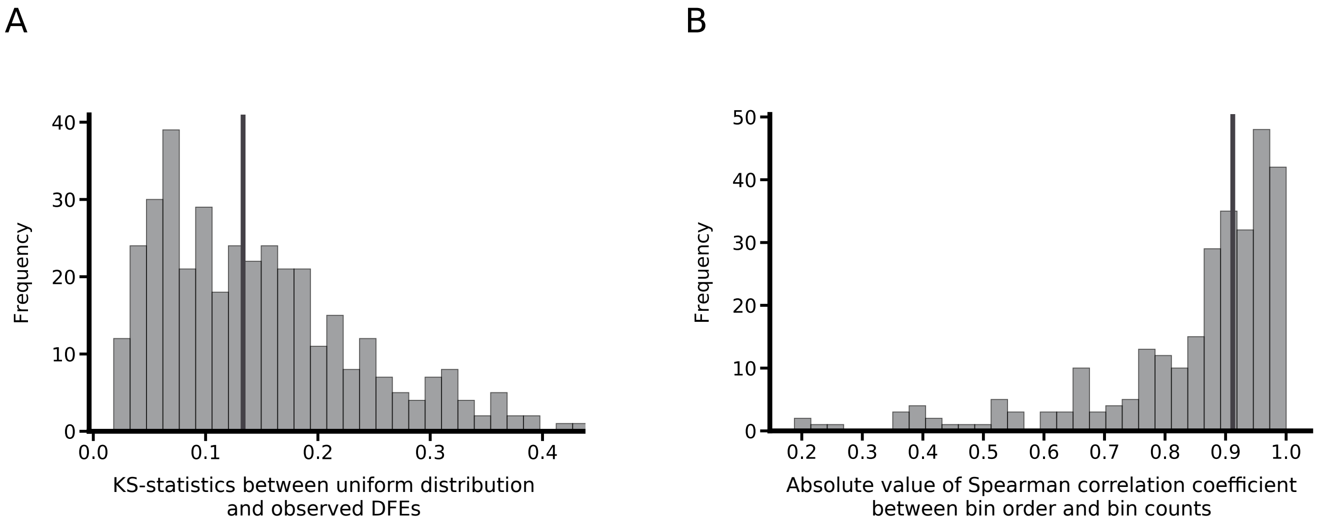

Fig. S2. Statistical characteristics of 380 distributions of fitness effects. (A) Most of the distributions are broad, characterized by relatively small deviations from uniform distributions measured by the KS-statistics (vertical line indicates the median). (B) Most of the distributions tend to be monotonic, indicated by the highly right-skewed Spearman correlation coefficients between the bin order and bin counts (vertical line indicates the median).

bioRxiv preprint doi: https://doi.org/10.1101/2024.10.11.617952; this version posted October 15, 2024. The copyright holder for this preprint (which was not certified by peer review) is the author/funder, who has granted bioRxiv a license to display the preprint in perpetuity. It is made available under aCC-BY 4.0 International license.

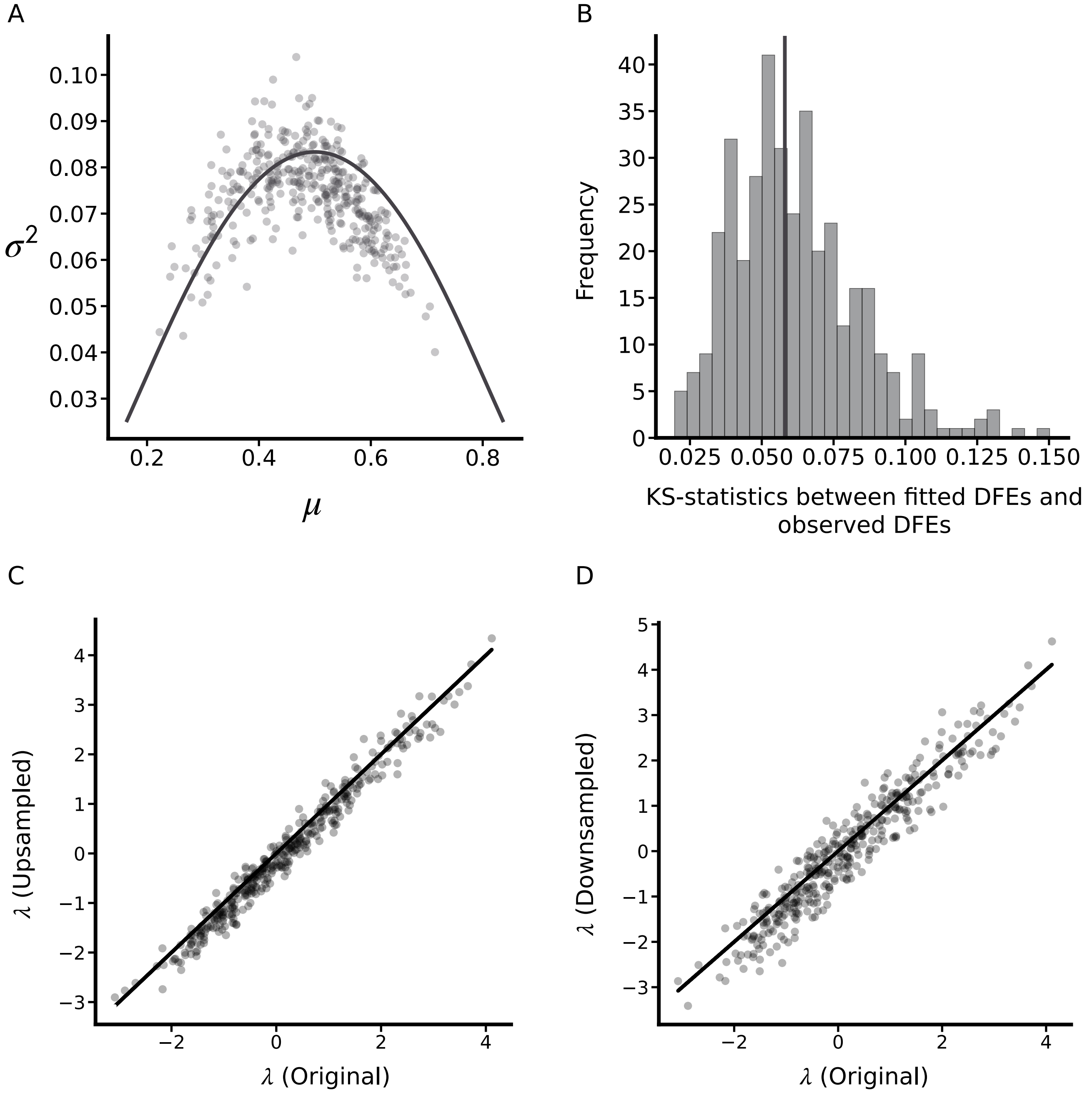

Fig. S3. Truncated exponential captures the 380 DFEs well. (A) The theoretical relationship (solid line) between expectation and variance of truncated exponential distributions qualitatively matches the observed data. (B) The KS-statistics between fitted truncated exponential distributions versus individual DFEs tend to be very small (vertical red line indicates the median), suggesting that truncated exponential distribution provides a good fit to the data. (C) and (D): the λ values estimated by equalizing the representation of each type, either by upsampling to the maximum count of all mutation types in the original dataset (C), or by downsampling to the minimum (D), is highly correlated to the original estimation, indicating that the truncated exponential distribution observed is not an artifact of unequal distribution of amino-acids in natural proteins (solid line indicates $y = x$).

bioRxiv preprint doi: https://doi.org/10.1101/2024.10.11.617952; this version posted October 15, 2024. The copyright holder for this preprint (which was not certified by peer review) is the author/funder, who has granted bioRxiv a license to display the preprint in perpetuity. It is made available under aCC-BY 4.0 International license.

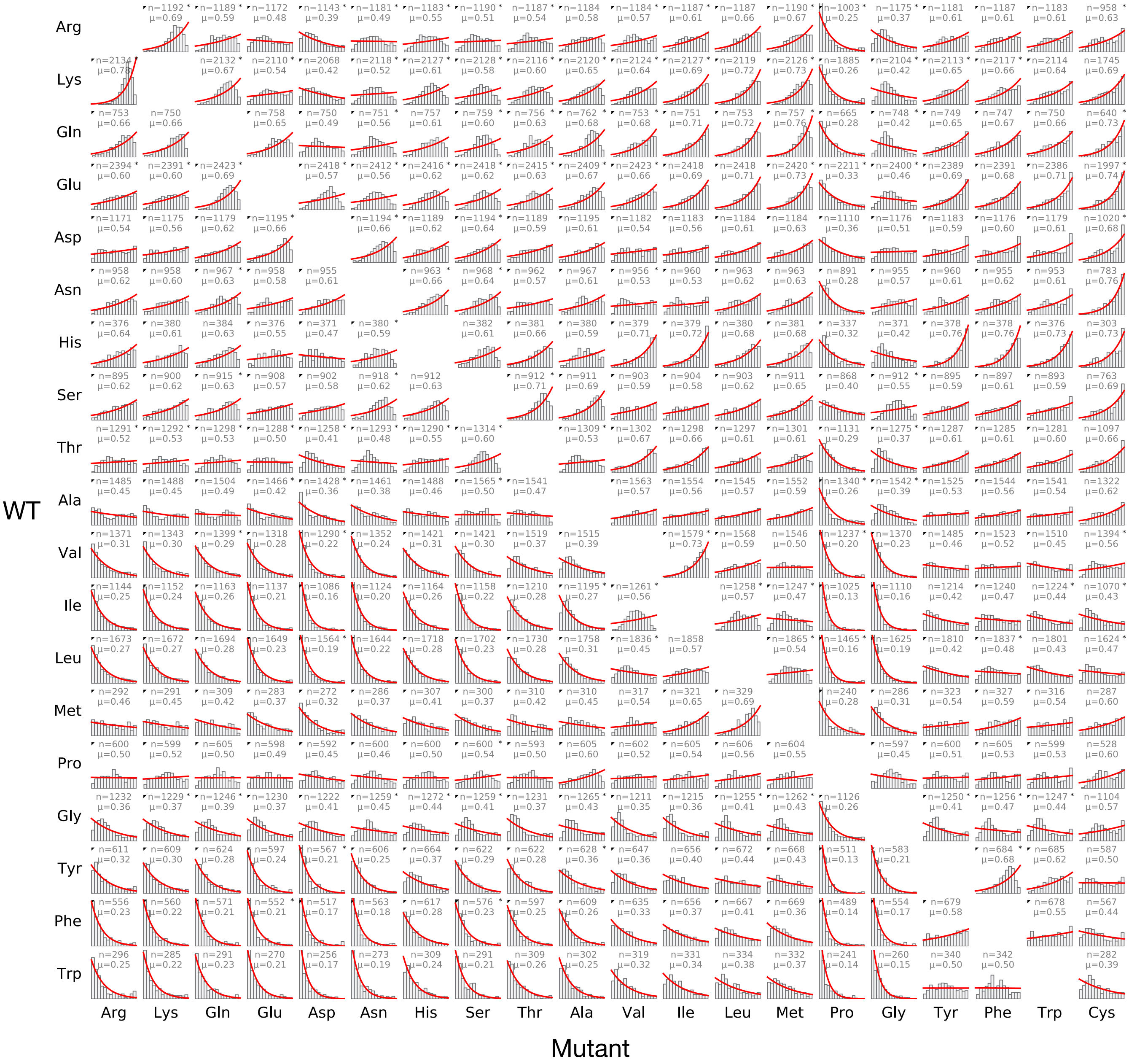

Fig. S4. Distributions of mutational effects on protein-folding for 389,030 amino-acid-altering mutations, categorized by the wild-type (row) and mutant (column) amino acids. Histograms illustrate the observed fitness effect distributions for mutations relative to each amino acid type, along with the truncated exponential fit for each distribution (red lines). Distributions that significantly deviate from a truncated exponential distribution are marked with a star at the right corner, whereas black triangles in the upper left corner indicate the pairs with significant forward-reverse asymmetry (Bonferroni-corrected $p < 0.05$, two-sample Kolmogorov–Smirnov test). For each distribution, the mean, which is equal to the probability that a replacement of this type is fitter than a random mutation, is given by μ , while the number of observations is given by n.
